# Decellularized Tendon Extracellular Matrix—A Valuable Approach for Tendon Reconstruction?

**DOI:** 10.3390/cells1041010

**Published:** 2012-11-05

**Authors:** Gundula Schulze-Tanzil, Onays Al-Sadi, Wolfgang Ertel, Anke Lohan

**Affiliations:** Department of Orthopaedic, Trauma and Reconstructive Surgery, Campus Benjamin Franklin, Charité-University of Medicine Berlin, Garystrasse 5, Berlin 14195, Germany; Email: onays.al-sadi@charite.de (O.A.-S.); wolfgang.ertel@charite.de (W.E.); anke.lohan@charite.de (A.L.)

**Keywords:** tendon healing, tenocytes, extracellular matrix, decellularization, recellularization

## Abstract

Tendon healing is generally a time-consuming process and often leads to a functionally altered reparative tissue. Using degradable scaffolds for tendon reconstruction still remains a compromise in view of the required high mechanical strength of tendons. Regenerative approaches based on natural decellularized allo- or xenogenic tendon extracellular matrix (ECM) have recently started to attract interest. This ECM combines the advantages of its intrinsic mechanical competence with that of providing tenogenic stimuli for immigrating cells mediated, for example, by the growth factors and other mediators entrapped within the natural ECM. A major restriction for their therapeutic application is the mainly cell-associated immunogenicity of xenogenic or allogenic tissues and, in the case of allogenic tissues, also the risk of disease transmission. A survey of approaches for tendon reconstruction using cell-free tendon ECM is presented here, whereby the problems associated with the decellularization procedures, the success of various recellularization strategies, and the applicable cell types will be thoroughly discussed. Encouraging *in vivo* results using cell-free ECM, as, for instance, in rabbit models, have already been reported. However, in comparison to native tendon, cells remain mostly inhomogeneously distributed in the reseeded ECM and do not align. Hence, future work should focus on the optimization of tendon ECM decellularization and recolonization strategies to restore tendon functionality.

## 1. Introduction

**Figure 1:**
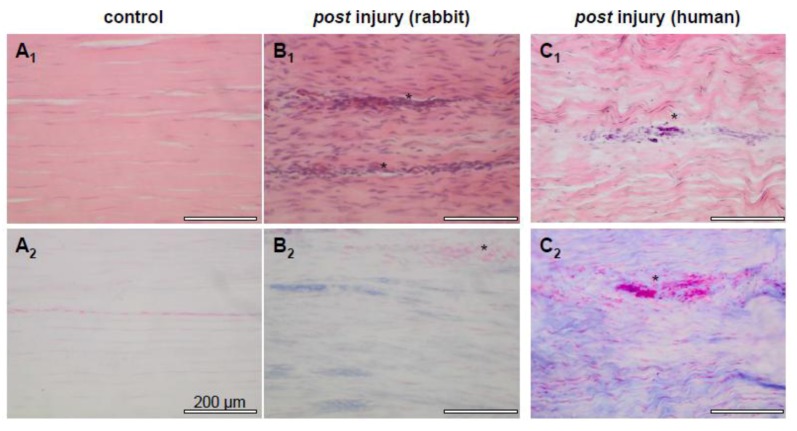
Tendon repair. Healthy rabbit Achilles tendon stained with haematoxylin eosin (HE) (**A_1_**). Elongated cell nuclei are visibly arranged in rows and surrounded by a dense ECM. For showing sulphated proteoglycans, alcian blue staining was performed (**A_2_**). The sulphated proteoglycans could hardly be detected. 6 weeks *post* injury (3 mm diameter tendon defect in the rabbit model), a hypercellularity with predominantly fibroblasts, an irregular and looser arrangement of ECM fiber bundles and multiple neo-vessels (asterisks) were evident (**B_1_**). In addition, a focal increase in proteoglycan deposition (**B_2_**) could be detected. Human Achilles tendon several weeks after rupture stained for HE (**C_1_**) and alcian blue (**C_2_**). Scale bars: 200 μm. Longitudinal sections are shown. Asterisks: neo-vessels.

Tendon injuries still remain an orthopedic challenge [1,2]. The time-consuming healing process extends over many months and usually leads to a reparative scar tissue [3,4] (Figure 1). Scars provide inferior biomechanical stability [5]. Multiple tissue engineering (TE) strategies have been developed in order to accelerate and improve tendon healing or to reconstruct tendons. Many biomaterials are currently being tested as implants for tendon reconstruction, among them synthetic polymers and natural ECM components such as poly-glycolic acid (PGA), poly-lactid glycolic acid (PLGA), polysaccharides such as chitosan or various collagen derivates [6,7,8,9,10]. These biomaterials have several shortcomings such as their limited biocompatibility, too rapid or hardly controllable degradation rates, biomechanical weakening during degradation and their low biofunctionality that limit the healing success of reconstructed tendon defects as reviewed by Liu *et al.* [6]. It must be noted that tenocytes require a long time to produce a dense, collagen-rich neo-matrix consisting of parallel aligned type I collagen fibers. In tendon repair tissue and tissue engineered constructs, type III collagen often dominates, which is of lesser strength compared with type I collagen [11]. Another strategy in tendon and ligament reconstruction is the use of autografts, such as the use of so-called hamstring tendons (*Musculus [M.] gracilis* and *M. semitendinosus*), for anterior cruciate ligament (ACL) reconstruction [12]. The biomechanical stability, adapting to the exact length, and achieving a stable fixation of the autograft, are all important requirements. Donor-site morbidity could, in some cases, limit the applicability of this technique. Therefore, allo- or xenogenic cell-free ECM has started to attract more and more attention in various fields of tissue reconstruction: complex parenchymal organs have already been decellularized and subsequently reconstituted with cells such as from the kidney, lung and liver, with the hope of utilizing them in the future for transplantation purposes [13,14,15,16]. Decellularization of the donor tissue is necessary to removing cellular antigens [17]. Since tendon reconstruction requires highly stable scaffolds, the implantation of a natural ECM might provide a promising therapeutic strategy. Hence, decellularized tissues such as porcine intestinal submucosa [18] and decellularized natural tendon ECM have started to attract much interest for tendon reconstruction [19,20,21,22]. For the treatment of rotator cuff tendon tears, xenogenic ECM derived from the human, bovine, equine or porcine dermis, intestinal submucosa or pericard, has already entered the clinical praxis [23,24,25]. These therapeutic approaches do not require the seeding of the ECM with autologous cells before implantation. However, the combination of the ECM with cultured autologous cells may improve the healing results. Although the use of a decellularized ACL-ECM instead of an autografted tendon could circumvent donor-site morbidity of autografting, it remains questionable whether decellularized ACLs are suitable for ACL reconstruction.

For this reason, it may be a promising approach to reconstruct damaged tendons using an allo- or xenogenic tendon ECM as a natural scaffold, reseeded with autologous human tenocytes or other more abundant or proliferative cell types that possess a tenogenic differentiation potential. Advantages of a natural ECM as a scaffold are its matching biomechanical properties as well as suitable biochemical and structural composition to guide cell growth. In addition, growth factors are entrapped within the natural ECM, which provides adequate tenogenic stimuli accelerating cell differentiation [26,27]. In light of this, this review summarizes and critically discusses currently published results of experimental settings based on decellularized ECMs for tendon reconstruction. 

## 2. Results and Discussion

### 2.1. Tendon and Ligaments

**Figure 2:**
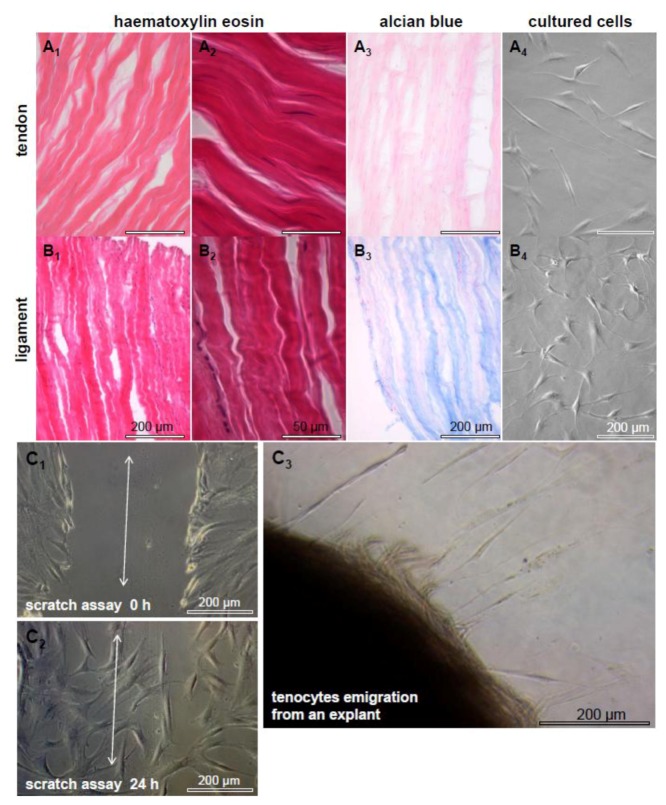
Tendon and ligament. Haematoxylin eosin (**A_1-2_**, **B_1-2_**) and alcian blue (**A_3_**, **B_3_**) stainings of a human hamstring tendon and an anterior cruciate ligament are shown. The cell nuclei of the ligament are more rounded, the ligament ECM has a more crimp appearance and contains more sulphated proteoglycans compared with the tendon. Longitudinal sections are shown. Scale bars: 200 μm (**A_1_**, **B_1_**, **A_3_**, **B_3_**, **A_4_**, **B_4_**), 50 μm (**A_2_**_, _**B_2_**). Isolated and cultured cells of the tendon (**A_4_**) and cruciate ligament (**B_4_**) are depictured. Cells of both tissues form multiple cell–cell contacts in culture. Migrating human tenocytes in response to a scratch in the cell layer (**C_1_** double head arrow), tenocytes fill the gap within 24 h (**C_2_**). Tenocytes, which migrate from a tendon explant 1 week after starting the culture, are shown (**C_3_**). Scale bars: 200 μm.

In order to reconstruct tendon defects by novel TE techniques it is important to understand the peculiarities of these tissues. Both tendons and ligaments are bradytrophic and hypovascular tissues composed of a few cells which are embedded within an abundant ECM consisting of mostly parallel arranged dense collagen fiber bundles and a few elastic fibers [28]. Ligaments connect two bony attachment points and tendons transmit forces from the muscle belly via their (in the most cases) fibro-cartilaginous enthesis to the bone. Only 5% of the normal tendon tissue volume is represented by resident cells in the tendon, mostly tenocytes which are a highly specialized fibroblast population. However, 95% of the tendon volume consists of ECM [29]. Some inconspicuous histological and biochemical differences exist between tendons and ligaments, as already previously reported [30,31,32]. A more crimp pattern of the ECM and a more ovoid shape of the cell nuclei can be observed in the human anterior cruciate ligament when compared with a hamstring tendon [3] (Figure 2). Additionally, the ECM composition, fiber cross-linking patterns and fibroblast responses, such as their remodeling capacities, differ between ligaments and tendons, depending on their localization and their particular mechanical requirements [3,33]. The ECM composition of tendons can be altered under pathological conditions [34,35]. Particularly the proteoglycan and elastic fiber contents differ in healing or degenerating tendons [9,36,37,38,39]. The enthesis, midsubstance and myotendinous junction regions of one tendon reveal a unique ECM composition [36,37,38,39,40]. Tendon ECM contents, the numbers of collagen as well as elastin cross-links and the fiber diameters vary also, depending on the maturation state of one individual [39,41,42]. Elastin fibers are cross-linked by lysyl oxidase in a similar manner like collagen fibers [43]. Thus, a higher number of cross-links increases the stability of elastic fibers. Interspecies differences concerning the tendon ECM composition, cellularity, tenocyte morphology and characteristics should be considered in view of xenogenic tendon or ligament ECM transplantation [44]. Hereby, the individual capacity of each tendon to sustain tensile load should be calculated and it has to be shown which tendon is appropriate for which particular application. On the whole, the choice of a suitable ECM donor tendon for the reconstruction of a particular injured tendon requires attention. In addition, the tendon-derived fibroblasts harvested for autologous implantation should be critically considered since tenocytes might possess “a positional memory” [33].

Tenocytes represent the majority of resident cells in tendons (Figure 2) and are a source for intrinsic tendon healing [45]. They are capable of producing all components of a mature tendon and they organize the tendon ECM fiber bundles consisting of densely packed parallel aligned collagen. Between them are 1%–2% elastic fibers, responsible for the wavy structure of the relaxed tendon and mediating its reversible stretchability under tension [28]. The elastin content permits tendon elasticity, whereby increasing elastin amounts mostly go hand in hand with reduced ECM stiffness [46].

Tenocytes lie in rows embracing bundles of collagen with their cytoplasmic extensions, whereby they are interconnected by gap junctions consisting of connexins 43 and 32 [47]. Connexins may synchronize the tenocytes within and between the cell rows of a tendon for effective mechanotransduction and adequate coordinated responses to stretching [48,49]. In response to an altered or enhanced tensile loading, tenocytes remodel and adapt the tendon ECM by the release of degradative enzymes and neo-synthesis of ECM components [50,51]. Their intimate ECM contacts are mediated by integrins as a major group of multifunctional cell-ECM cell surface receptors which control the ECM homeostasis and *vice versa* for cell adherence, differentiation and survival [50,51]. Despite the fact that they form multiple cell–cell contacts *in vitro*, cultured tenocytes and ligament cells usually do not align in rows (Figure 2), thereby suggesting that either biomechanical forces or the strictly parallel-arranged ECM bundles induce the cell alignment. *In vitro* studies revealed that an aligned microtopology of biomaterials can promote tenocytes’ alignment [26,52]. Nevertheless, it remains an unanswered question whether the functionally important cell-cell and cell-ECM communication can be adequately restored in reconstructed tendons consisting of a decellularized and reseeded xenogenic tendon ECM.

### 2.2. Tendon Healing and Tendon Reconstruction

Intrinsic and extrinsic healing processes can be distinguished during tendon repair [3,53,54]. Healing can occur intrinsically, by proliferation of the tenocytes, or extrinsically, by invasion of cells from the tendon environmental tissues [45,53,54]. Thus, extrinsic healing comprises the immigration of cells from the tendon sheath, tendosynovium and fascia often accompanied by the problem of tendon adhesion formation and, subsequently, hindered tendon gliding [45,55,56]. It usually leads to scar tissue with less biomechanical strength [5]. Intrinsic healing is characterized by the proliferation of intrinsic cells such as epi- and endotenon tenocytes [45,57,58]. Less scar tissue formation and better results mostly lacking adhesion formation can be observed, as compared to the extrinsic healing processes [45,57,59]. In most cases, the tendon defects cannot be adequately reconstructed by the healing response [3]. For this reason, tendon TE could be a suitable strategy to provide implantable neo-tissues based on scaffolds suitable to guide cells’ entry, promote their proliferation, distribution and alignment, and altogether resulting in an accelerated tendon healing.

**Figure 3:**
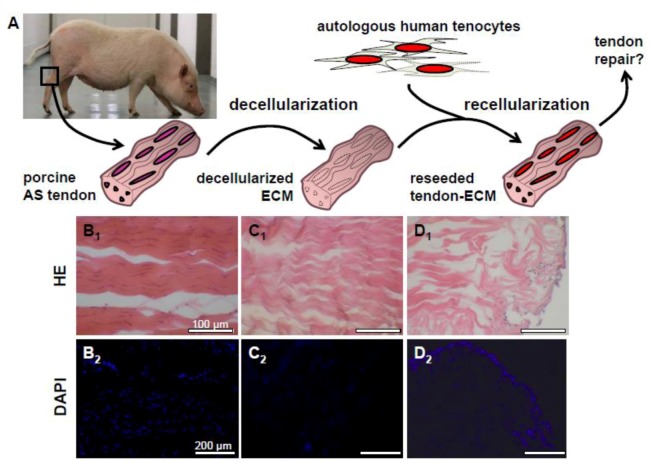
Dezellularization and recellularization of porcine Achilles tendons for tendon repair.Schematic diagram of the decellularization of porcine Achilles (AS) tendon as a scaffold for reseeding with autologous human hamstring tenocytes to use the resulting constructs for tendon defect coverage (**A**). HE and 4',6-diamidino-2-phenylindole (DAPI) staining of a native (**B_1_**, **B_2_**), decellularized (**C_1_**, **C_2_**) and a porcine Achilles (AS) tendon ECM recolonized with human hamstring tenocytes (**D_1_**, **D_2_**). Clefts arise between collagen fiber bundles in response to decellularization. Scale bars: 100 µm (**B_1_, C_1_, D_1_), **200 μm (**B_2_, C_2_, D_2_)**.

Autografts such as hamstring tendons, *M. plantaris* or *M. palmaris longus* tendons are often used for tendon and ligament reconstruction, whereby donor site morbidity remains a limiting criterion for autografting [60]. Arguments to use natural decellularized ECM to reconstruct tendons (Figure 3) are the abundant availability of allo- or xenogenic tissues bypassing donor site morbidity and the fact that the ECM serves as an optimal structural, biochemical and biomechanical template for neo-tendon formation. In view of harvesting xenogenic tissues for transplantation purposes, the elimination of immunogenic antigens is strongly required. The fact that these antigens are mostly cell associated demands a complete decellularization of the tissue. 

### 2.3. Decellularization of Tendons

Several decellularization strategies, suitable for removing antigenic cellular components, have been developed. Most of them are based on repeated freezing, vigorous mechanical agitation and of the use of typical detergents such as triton X-100, sodium dodecyl sulphate (SDS), sodium desoxycholate, chelating agents, e.g., ethylenediaminetetraaceticacid (EDTA), zwitterionic detergents, such as 3-[(3-Cholamidopropyl)-dimethylammonio]-1-propansulfonat (CHAPS), alkalines or acids e.g., peracetic acid, or various enzymes such as trypsin and nucleases, alone or in combination [61,62,63,64]. Deeken *et al.* recommended particularly the zwitterionic detergent tri(n-butyl)phosphate for decellularization, which was able to completely eliminate the cell nuclei, whereas the ECM structure was mainly conserved [63]. SDS, as an ionic detergent, destroys the cell membrane and denatures proteins and for this reason, affects the collagen structure; triton X-100 is a nonionic detergent, which hinders the lipid–lipid and lipid–protein interaction, but extracts also some glycosaminoglycans (GAGs) from the ECM; the enzyme trypsin splits the peptide’s binding of arginine and lysine, but removes also laminin, fibronectin, elastin and GAGs from the ECM [61]. These various decellularization strategies have already been summarized and thoroughly discussed [61,63,64,65]. The leftovers of cell nuclei or other cellular components are immunogenic and can lead to macrophage activation. Activated macrophages might induce scaffold remodeling [66]. Hence, controlling the decellularization success is of major importance and can be done simply by histological stainings e.g., haematoxylin eosin, e.g., combined with DNA content measurements [19], or various DNA stainings [19,62]. Nevertheless, it remains to be seen whether or not these techniques are really sensitive enough to detect immunogenic cellular remnants. Gui *et al.* proposed the use of fetal calf serum as an effective approach to remove remnants of DNA from the decellularized tissue [67]. In addition, the residues of decellularization buffers, such as the aforementioned detergents, might impair viability of colonizing cells and induce a sterile inflammation [61]. Thus, inflammatory reactions can occur when decellularized tendons are implanted [68]. In some studies, the decellularized ECM was very compatible *in vitro* and, if implanted *in vivo,* it did not exhibit a host-cell-mediated foreign-body immune response [69]. However, the study of Keane *et al.* clearly indicated that a poor decellularized ECM induced a macrophage M1 shift as a feature of an inflammatory host response [66].

A wash out of proteoglycans or GAGs and a loss of growth factors usually bound to the ECM can be caused by decellularization [61]. The washout of proteoglycans might lead to a loss of the coherence of collagen fiber bundles [61,70]. Despite its inferior biomechanical stability compared with a mature ECM, the use of juvenile, immature tissue bears the particular advantage in offering a stronger tenogenic impulse based on a higher content of specific tenogenic growth factors. Typical growth factors supporting tenogenic differentiation are the growth and differentiation factors (GDF) 5, 6 and 7 [71], fibroblast growth factor-2 (FGF-2) [5], and the bone morphogenetic protein (BMP)2 [72]. In addition, the transforming growth factor (TGF)-β1 promotes the healing response in tendon [73].

Further, decellularization can induce some loss of tendon ECM structure [61]. The ECM becomes denser due to the loss of laminin or fibronectin, whereby the cell immigration is hindered later during recolonization. In addition, the sterilization procedures for the decellularized ECM, such as those based on ethanol, can lead to a higher density of the ECM. Ultrasonication has been proposed as a strategy to loosen up decellularized tendons in order to facilitate cell infiltration [62]. A high number of handling and freezing cycles during decellularization impairs the biomechanical stability of the ECM [74]. Thus, a loss of tendon structure can be observed during decellularization (Figure 3B–3D), which usually goes along with some decrease in biomechanical competence [74]. In contrast, Pridgen *et al.* concluded that the biomechanical competence in decellularized flexor tendons was mainly maintained, since the resistance to ultimate tensile stress did not significantly differ between decellularized and native tendons [19]. 

### 2.4. Recellularization of Decellularized Tendon ECM

It is still up for debate whether the recellularization of an ECM is indeed necessary to prepare it for implantation. Cells seeded on the ECM remained detectable for several weeks *in vitro,* but cell numbers decreased [62]). Omitting the recellularization procedure may save time and also the biopsy, which is necessary for harvesting autologous cells for reseeding. Furthermore, it has been observed that most of the cells seeded onto the ECM scaffold were later (30 weeks *post* implantation *in vivo*) replaced by the immigrating host cells [75]. Tenocytes possess a high migratory capacity detectable *in vitro* (as shown in Figure 2C, a scratch in a cell layer is completely closed after 24 h). We observed that recellularized tendons are better colonized *in vivo* in the nude mice xenograft model when compared with the cell-free ECM alone. It might be caused by the fact that tenocytes somehow prime the ECM for cell colonization, most likely by deposing their own pericellular ECM enriched with growth factors which provide chemotactic signals attracting immigrating cells underlining the concept of dynamic reciprocity [76]. Biomechanical properties were improved in response to recellularization of the cell-free tendon ECM with tenocytes compared with cell-free implants [75].

Tenogenic and highly proliferative cells would be a suitable source for ECM recellularization. Various cell types have already been tested: in the majority of cases, tenocytes were chosen [22,75,77], representing the resident cell type in tendon. However, some cell types, which can be harvested in high cell numbers with only a low risk of donor site morbidity, such as mesenchymal or adipose tissue derived stem cells (MSC, ASC), tendon sheath fibroblasts [20,78] or dermal fibroblasts [19,68,74,79,80], have also been studied. Many of them are characterized by a higher proliferative activity. Moreover, Broese *et al.* used simply bone-marrow aspirates for reseeding of decellularized human tendon and found similar results when compared with the use of isolated MSCs [81]. These cell types, e.g. MSCs, can differentiate towards a tenogenic lineage when seeded into the tendon ECM [82]. The tendon ECM provides inductive impulses to progenitor cells engaging ligand/ECM receptors interaction and *vice versa* leading to cytoskeleton organization according to the concept of dynamic reciprocity [76]. Differentiation of progenitor cells requires additional time and essential tenogenic stimuli such as the supplementation with growth factors. Seeding a decellularized ECM with tendon cells and stimulating them with growth factors led to a higher ECM synthesis in recellularized tendons compared with the use of bone-marrow derived progenitor cells as reported by Durgam *et al.* [80]. It remains questionable whether or not tendon-derived cell types can adequately substitute tenocytes, which are well known to mediate intrinsic healing processes in injured tendons.

Major milestones in the recellularization procedures are to gain a homogeneous cell distribution and to achieve sufficient cell survival. The cell content in mature tendons is low [28]. During tenogenesis and tendon maturation, the cell content in the tendon continuously decreases. Therefore, immature tendons possess a higher cellularity than mature tendons, which contain only few cells [39,42]. Whether the cell numbers recommended for recellularization experiments of cell-free ECM, such as two million tenocytes per mL for 3-5 cm-long tendons [22,75], or a similar number of tendon-derived fibroblasts or stem cells for 1.5 cm-long rabbit flexor tendons [20] were indeed suitable, remains unclear. However, the exact cell suspension volume for the recellularization procedure was not mentioned by both cited research groups. Pridgen and Tischer *et al.* used two million dermal fibroblasts in one mL growth medium per one cm tendon [19,68].

Reseeding of the cell-free tendon ECM remains a challenge since the ECM is dense and does not allow sufficient cell infiltration [19]. Cells often remain at the ECM surface [22]. Various static [22,77] and dynamic seeding strategies [75,79,83] have been applied to improve recolonization of the tendon ECM [78]. It has to be recognized that cells immigrate predominantly from the tendon stumps into the ECM. Recently, it has been reported that tendon is surrounded by a basallamina-like structure [84], which might inhibit cell immigration from the lateral directions. Tendon ECM can be loosened up by ultrasound sonication and, additionally, by splitting the cell-free tendons into 500 μm-thick fascicular scaffolds [62]. Peracetic acid treatment of decellularized tendons improved their porosity [79]. Cells can be injected into tendon ECM [85] using a gauge; however, some cell and tissue damage has to be expected as induced by this technique [86]. Cells can also be fixed within the scaffolds using fibrin glue or an alginate hydrogel [85]. Petri *et al.* established a continuous perfusion setting for seeding tendon ECM which obviously improved the growth of MSC within a decellularized Achilles tendon ECM when compared with static seeding conditions [78].

### 2.5. *In Vivo* Studies Using Decellularized Tendon

Until now, most of the published studies about recellularized tendon ECM reflect *in vitro* results. However, for the treatment of rotator cuff tears, cell-free xenogenic mammalian tissues have been used for several years, although further optimization is still demanded [23,24,25]. One critical point is to improve cell infiltration into the implanted ECM scaffold. Most of the ECMs selected for this purpose do not derive from tendons, but are based on other collagen-rich tissues such as the dermis, intestinal submucosa and the pericard. The collagen fiber architecture of these ECMs consisting of crossing fiber bundles differs from the tendon. The remodeling process, which starts in response to implantation, requires substantial time periods depending on tissue origins and collagen cross-linking patterns, and it leads to divergent outcomes [25]. The degradation of the decellularized tissue following implantation typically results in a loss of mechanical properties while the rebuilding of the scaffold as a time-dependent process of remodeling is dependent upon many factors, including mechanical loading (rehabilitation). Therefore, success or failure may depend upon factors other than the decellularized tissue itself.

Achievement of blood supply and host tissue integration could represent critical points for these scaffolds. Chen *et al.* investigated rotator cuff tendon repair in rabbits using cell-free porcine intestinal submucosa, whereby these scaffolds, seeded with autologous tenocytes, led to a better healing and remodeling response than the implantation of the bioscaffolds alone, particularly the hosts’ inflammatory response was substantially reduced [18]. One might therefore hypothesize that the ECM fibers derived from the foreign donor can be covered by an autologous ECM produced by the cells seeded onto the ECM.

MSCs seeded onto decellularized canine tendons and implanted in rabbit patellar tendon defects exhibited a tendon-like phenotype characterized by a higher expression of the tendon marker tenomodulin in the implants containing BMSCs, as compared to the cell-free implants *in vivo,* underlining the tenogenic stimulus of a cell-free tendon ECM [82]. Kryger *et al.* filled decellularized rabbit tendons seeded with tenocytes or tendon sheath fibroblasts, bone marrow MSCs, or ASCs in defects of the *M. flexor digitorum profundus* tendon using a rabbit model [20]. The research group observed that cells of all cell types tested in their study remained still viable for at least six weeks after *in vivo.* They could still be detected lying within the tendon architecture. Cell-free tendons have been implanted in nude or CD1 mice, whereby their high biocompatibility became evident. The tendon ECM was readily infiltrated by the host cells and it mainly retained its biomechanical competence [69]. Raghavan *et al.* implanted decellularized flexor tendons into Wistar rats which were fixed to the spinal ligaments and found a removal of cellular antigens and better biomechanics compared with the control tendons [17]. Nevertheless, the question should be further addressed whether the remodeling of the ECM construct *in vivo* is sufficient to produce a reparative tissue able to fully withstand natural mechanical strain and to gain access to blood and oxygen supply. 

### 2.6. Blood Vessel Access in Tendon

The tendon is usually a hypovascularized bradytrophic tissue able to sustain low oxygen tensions [87,88]. Only 1%–2% of the tissue consists of blood vessels [50,89]. Superficial vessels follow the tendon sheath and paratenon connective tissues to get via the endotenon into the tendon; other vessels penetrate the tendon from the myotendinous and enthesis site, whereby these vessels do not reach the middle-third of the tendon, which, in most cases, leads to hypovascular areas [45,88,89,90,91]. Hence, in the midsubstance of healthy tendons, usually only a few small capillaries are detectable. Cyclic loading in these areas leads to ischemic stress. These regions are therefore often the starting point for tendon ruptures and degenerative alterations [88,89]. Intrasynovial tendons, such as flexor tendons of the fingers or intraarticular tendons and ligaments, like the long biceps tendon or cruciate ligaments, gain some nutrient supply via synovial fluid [92]. Under hypoxic conditions, for example during tendon healing, large diameter blood vessels chiefly at the myotendinous junctions, but also in the midsubstance of the tendon and in the epitenon, appear as a correlate of increased angiogenesis [3,9] (Figure 1). It is unclear whether an implanted tendon ECM can be sufficiently revascularized *in vivo*. Initial hypoxia, detectable before the implant accesses blood supply, could lead to the loss of the colonizing cells. For this reason, future strategies should also take into account the construct’s vascularization, whereby, for instance growth factor supplementation and the addition of endothelial cells, may be beneficial. Vascular endothelial growth factor (VEGF) is a candidate which is upregulated in tenocytes under hypoxic conditions and promotes endothelial cell proliferation and, consequently, angiogenesis [93]. Additionally, myodulin is expressed in tenocytes [94]. It modulates blood vessel formation in the muscle and at the myotendinous junction, probably by interacting with endothelial cell proteins [95]. Several experimental studies revealed that blood platelet extracts such as platelet rich plasma (PRP) can improve tendon healing [96,97,98,99]. However, controversy remains, whether the observed effects are indeed of clinical relevance [100,101]. The effect of platelet extracts is based on the release of various anabolic and angiogenic growth factors (TGF-β1, VEGF, platelet derived growth factor [PDGF]) as well as other factors such as thrombospondin-1 and the ECM components fibronectin and vitronectin [96]. Another study indicated that PDGF upregulated tenocytes’ VEGF expression [102]. Hence, platelet extracts could offer an approach to accelerate cell immigration into decellularized tendons and their neo-matrix synthesis. The successful access to blood supply by decellularized tendon implants has not been reported yet. 

### 2.7. Lymphatic Vessels and Tendon Regeneration

In contrast to blood vessels, more lymphatic vessels cover the surface of tendons [103,104,105] and some of them penetrate into the endotenon between the primary fascicles of the tendon [106]. The lymphatic drainage is important for interstitial fluid balance and immune cell trafficking [107]. Lymphatic vessel regeneration might also contribute to the individual healing outcome of tendons. Whether there exists an essential cross talk between tenocytes and lymphatic endothelial cells supporting tendon regeneration remains unclear. Some attempts have been undertaken to recellularize acellular tissues with endothelial cells to tissue engineer blood vessels [108], and recently, Niklason *et al.* proposed a similar procedure to engineer lymphatic vessels [109]. 

### 2.8. Novel Approaches: Tenocytes Co-Culturing with Endothelial or Other Cells

It is well known that within most tissues, and probably also in tendon, several cell types communicate with each other. Although the majority of resident cells in tendons are tenocytes, also some endothelial and probably smooth muscle cells derived from blood capillaries and small lymphatic vessels can be detected [110]. Further, synovial cells can be found in intrasynovial tendons, such as many flexor tendons if they immigrate during healing into the tendon [54]. Fibro-chondrocytes can be observed in the enthesis region and, in some tendons’ avascular areas containing fibro-cartilage, they are formed to sustain pressure load [89]. Therefore, the question arises whether a co-culture setting consisting of a majority of tenocytes with a minority of endothelial or other aforementioned cell types might improve the recellularization results of acellular ECM and accelerate blood vessel access after implantation. Co-culture approaches of endothelial cells and smooth muscle cells were suitable in the reseeding of other cell-free tissues, such as decellularized heart valves [111,112]. Comparable to tendon, heart valves are mostly avascular tissues with a collagen-rich ECM. The effect of synovial fluid components on tenocyte ECM recolonization remains unclear and might be particularly interesting for reseeding of intrasynovial flexor tendons.

## 3. Limitations and Future Directions

The critical challenge in utilizing decellularized natural ECMs for tendon reconstruction (Figure 3A) is to develop mild decellularization strategies which maintain ECM structure, composition, and biomechanics, but allow complete removal of tissue donor cell components and good recellularization results. However, in comparison to native tendon in reseeded tendon ECM, the cells remain mostly inhomogeneously distributed with cell-free areas, and they usually do not align into rows [20,68] (Figure 3B–3D). For this reason, it should be further tested whether mechanical stimulation during reseeding can promote cell alignment. Immunogenicity and transmission of diseases could limit the application of xeno- or allogenic ECM, but effective decellularization and safe sterilization procedures circumvent these problems. A combination of natural ECMs with particular tenogenic growth factors, platelet extracts or designed polymer scaffolds might provide novel applications. Further, the combination of tendon-derived cell types in co-culture with tenocytes could improve the recellularization efficacy.

## 4. Conclusions

First, preclinical *in vivo* results of implanted recellularized tendons are encouraging. Compared with other scaffolds, cell-free tendon ECM provides a natural structural, biochemical and biomechanical template for neo-tendon formation. Future work should focus on optimization of tendon ECM decellularization and to develop effective cell recolonization strategies for obtaining tendon functionality.
